# A Master Medalist, a President, Tuberculosis, and a Congress: Contributions More Lasting than Bronze

**DOI:** 10.3201/eid2103.AC2103

**Published:** 2015-03

**Authors:** Terence Chorba

**Affiliations:** Centers for Disease Control and Prevention, Atlanta, Georgia, USA

**Keywords:** art science connection, emerging infectious diseases, coins, Lincoln, Roosevelt, art and medicine, Victor D. Brenner, International Congress on Tuberculosis Medal, 1908, A Master Medalist, a President, Tuberculosis, and a Congress: Contributions More Lasting than Bronze, tuberculosis, about the cover

**Figure:**
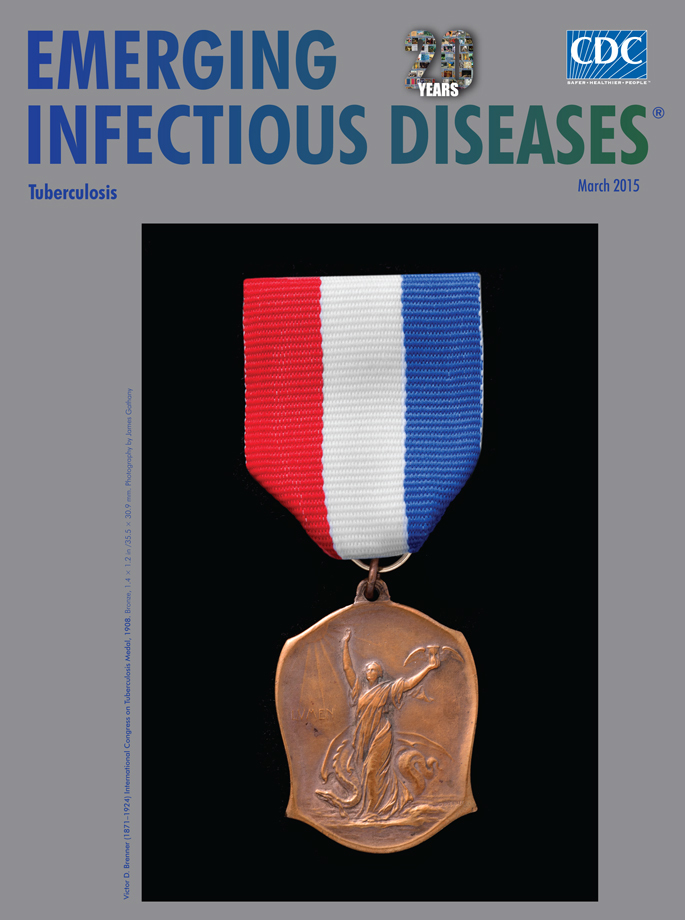
**Victor D. Brenner (1871–1924) International Congress on Tuberculosis Medal, 1908. **Bronze, 1.4 × 1.2 in /35.5 × 30.9 mm. Photography by James Gathany

The initials VDB are well known to collectors of the Lincoln penny, the obverse (front surface) of which reflects the longest-running design in the history of US coinage. In 1909, in its first coinage, VDB appears in embossed format at the bottom of the coin’s reverse, in honor of its designer, Victor David Brenner. The initial San Francisco Mint coin, the 1909-S VDB, remains a sought-after rarity; most collectors never fill the open-mouthed, empty “1909-S VDB” hole in their penny albums.

Despite a tradition of artists’ signing their names or initials in coinage, controversy arising from the prominence of the VDB initials resulted in their removal that same year. Tucked into the left lowermost ridge of the bust of Abraham Lincoln, Brenner’s initials were restored to the obverse of Lincoln pennies coined since 1918. Discernable by magnification, the inconspicuous placement of Brenner’s signature imitates that of a Sicilian engraver, Euainetos, whose work Brenner described as having an “extraordinary decorative sense woven into every line, giving to the empty spaces as much charm as to the modeled surfaces.”

Brenner, born Viktoras Baranauskas in Lithuania in 1871, immigrated to the United States in 1890, bringing with him stone-cutting skills that he had learned from his father. Employed as an engraver in New York City, he took evening classes first at Cooper Union, then at the National Academy School and the Art Students League of New York. In 1898, he began to study metal sculpting in Paris under Louis-Oscar Roty and Alexandre Charpentier and became acquainted with Auguste Rodin. Roty taught Brenner the lost wax method of casting to make medals. Brenner’s work subsequently won high honors at the Paris Exhibition (1900), Pan American Exposition in Buffalo (1901), and St. Louis International Exposition (1904).

After returning to New York City in 1906, Brenner produced a medal, plaquettes, and a bas-relief for the 1909 centenary of Lincoln’s birth. US President Theodore Roosevelt saw examples of those works while posing for Brenner during the making of a Panama Canal service medal, and Brenner gained two more commissions as a result. The first commission was to design a penny featuring Lincoln, a radical departure from tradition—heretofore, no president had appeared on US coins intended for widespread circulation. The second commission was to design a medal for the Sixth International Congress on Tuberculosis, to be held in Washington, DC, in 1908. Roosevelt, who was invited to preside over this Congress, wrote: “The importance of the crusade against tuberculosis… cannot be overestimated when it is realized that tuberculosis costs our county two hundred thousand lives per year, and the entire world over a million lives per year, besides constituting a most serious handicap to material progress, prosperity, and happiness, and being an enormous expense to society, most often to those in walks of life where the burden is least bearable.”

The National Association for the Study and Prevention of Tuberculosis (founded by Edward Trudeau in 1904 and now known as the American Lung Association) planned the Congress. Honorary presidents of the Congress included Trudeau and Robert Koch, one of the fathers of modern microbiology, who had described the causative agent of tuberculosis (TB) 25 years earlier. Many legendary figures in the United States and foreign medical communities participated, including William Welch (whose image was subsequently immortalized on a Brenner plaque at Johns Hopkins University), Hermann Biggs, William Osler, Arnold Klebs, and Charles Mayo. Albert Calmette also discussed the success that he, together with Camille Guérin, had achieved in immunizing cattle against TB by using an attenuated strain of *Mycobacterium bovis*. The relatively recent breakthroughs of Koch and of Calmette and Guérin offered hope that science would soon defeat TB.

The Congress was convened in what is now the National Museum of Natural History. For the time, it was a colossal meeting: the number of delegates exceeded 5,000, and total attendance at the various sessions was nearly 100,000. Each delegate received a bronze medal (this month’s cover image) designed by Brenner. On the obverse is the figure of a beautiful woman holding the hourglass of time and striding toward a radiant sun, under which is written the Latin word for light, “LVMEN.” The woman represents human scientific endeavors approaching enlightenment, and as she progresses, she is trampling down an evil dragon representing disease. On the reverse is the American eagle, with the stars and stripes; underneath appears “INTERNATIONAL CONGRESS ON TVBERCVLOSIS WASHINGTON 1908” and the double-barred cross, the insignia of the campaign against TB.

At the closing of the Congress, several adopted resolutions described the underpinnings of today’s federal, state, and local TB control programs. The first such resolution was as follows: “That the attention of the state and central governments be called to the importance of proper laws for the obligatory notification, by medical attendants, to the proper health authorities, of all cases of tuberculosis coming to their notice, and for the registration of such cases, in order to enable the health authorities to put in operation adequate measures for the prevention of the disease.”

This Congress justifiably captured the attention of those in medicine and government. In 1908, the US death rate from TB was estimated to be 164 deaths per 100,000 population, nearly 10% of deaths from all causes. For those who marvel at the vision of Roosevelt, the challenges remain: TB still claims more than a million lives annually worldwide and still disproportionately affects “those in walks of life where the burden is least bearable.”
